# Supplementary material for: Epidemiology and biology of a herpesvirus in rabies endemic vampire bat populations
Source: Nat Commun. 2020 Nov 23;11:5951. doi: 10.1038/s41467-020-19832-4 (PMC7683562; doi:10.1038/s41467-020-19832-4)
Supplement: Supplementary file 1 — Supplementary Information [file 41467_2020_19832_MOESM1_ESM.pdf]

Supplementary materials for:

Epidemiology and biology of a herpesvirus in rabies endemic vampire bat populations

Megan E. Griffiths[1,2*], Laura M Bergner[1,2], Alice Broos[1,2], Diana K Meza[1,2], Ana da Silva Filipe[1], Andrew Davison[1], Carlos Tello[3,4], Daniel J. Becker[5] and Daniel G. Streicker[1,2*]

This file contains:

- Supplementary Tables 1 – 7
- Supplementary Figures 1 – 2

Supplementary Table 1 – Binomial confidence intervals for bat species BHV prevalence estimates.

Species	Positive	Negative	mean (bayes)	95% lower CI	95% upper CI
Anoura geoffroyi	0	8	0.05555556	0	0.20750804
Artibeus fraterculus	0	5	0.08333333	0	0.30574558
Artibeus lituratus	1	2	0.375	0.009556873	0.77075725
Artibeus obscurus	0	4	0.1	0	0.36248681
Artibeus planirostris	0	2	0.16666667	0	0.56925853
Carollia perspicillata	0	43	0.01136364	0	0.04343663
Chiroderma salvini	0	2	0.16666667	0	0.56925853
Chiroderma trinitatum	0	1	0.25	0	0.77148019
Choeroniscus minor	0	1	0.25	0	0.77148019
Desmodus rotundus	124	4	0.96511628	0.933286891	0.99234526
Diphylla ecaudata	1	1	0.5	0.060830276	0.93916972
Glossophaga soricina	4	1	0.75	0.43601728	0.99829
Lonchorhina aurita	0	7	0.0625	0	0.23246478
Myotis oxyotus	3	8	0.29166667	0.065168374	0.53723936
Phyllostomus discolor	0	1	0.25	0	0.77148019
Rhinophylla pumilio	0	1	0.16666667	0	0.56925853
Rhynchonycteris naso	1	0	0.75	0.228519814	1
Saccopteryx bilineata	1	0	0.75	0.228519814	1
Sturnira lilium	2	4	0.35714286	0.054184371	0.68017254
Vampyressa bidens	0	4	0.1	0	0.36248681

Supplementary Table 2 – Sample sizes of each species tested for BHV by PCR, by sample type and species.

Species	Sample type	Number tested	Number sequenced
Anoura geoffroyi	Saliva/fecal	8	0
Artibeus fraterculus	Saliva	5	0
Artibeus lituratus	Saliva	3	1
Artibeus obscurus	Saliva	4	0
Artibeus planirostris	Saliva	2	0
Carollia perspicillata	Saliva/fecal	43	0
Chiroderma salvini	Saliva	2	0
Chiroderma trinitatum	Saliva	1	0
Choeroniscus minor	Saliva	1	0
Desmodus rotundus	Saliva	128	14
	Blood	43	0
Diphylla ecaudata	Saliva	2	1
Glossophaga soricina	Saliva	5	4
Lonchorhina aurita	Saliva	7	0
Myotis oxyotus	Saliva/fecal	11	3
Phyllostomus discolor	Saliva	1	0
Rhinophylla pumilio	Saliva	2	0
Rhynchonycteris naso	Saliva	1	1
Saccopteryx bilineata	Saliva	1	1
Sturnira lilium	Saliva	6	1
Vampyressa bidens	Saliva	4	0

Supplementary Table 3 – Summary of GLMM fixed effects (BHV_positive ~ Sex + Age + Structure + Ecorange + Year + Season + (1|Site))

	Estimate	Std. error	z value	Pr(>|z|)
(Intercept)	-4.81E+03	1.98E+04	-0.243	0.8079
SexM	6.60E-02	1.14E+00	0.058	0.9539
AgeJ	8.64E-01	1.14E+00	0.759	0.4481
AgeSA	3.62E+02	1.68E+07	0	1
StructureU	6.09E-01	1.45E+00	0.419	0.6752
EcoregionAndes	-1.96E+01	1.96E+04	-0.001	0.9992
EcoregionCoast	-2.19E+01	1.96E+04	-0.001	0.9991
Year	2.40E+00	1.44E+00	1.66	0.0969
Season	-8.52E-02	2.85E-01	-0.299	0.7647

Supplementary Table 4 – Individual link support in nucleotide and amino acid cophylogenetic analyses. Those which fall below the mean value and therefore are well supported, have been highlighted.

Host	Virus (Nucleotide sequence)	Jackknifed squared residuals
Myotis_oxyotus	'MyotisoxyotusCAJ1BHV'	0.320184
Miniopterus_schreibersi	'EF151197.1MiniopterusschreibersiiBHV1'	0.050065
Saccopteryx_bilineata	'SaccopteryxbilineataLR4BHV'	0.056766
Rhynchonycteris_naso	'RhynchonycterisnasoLR4BHV'	0.056766
Sturnira_tildae	'SturniraLR4BHV'	0.275508
Tadarida_teniotis	'JX294564.1TadaridateniotisBHV2'	0.106568
Diphylla_ecaudata	'DiphyllaecuadataBHV'	0.201146
Tupaia_picta	'AF281817.1TupaiaHV2'	0.313678
Cavia_porcellus	'HG531783.1CaviidHV2'	0.415217
Glossophaga_soricina	'GlossophagasoricinaLR4BHV1'	0.184372
Desmodus_rotundus	'DesmodusrotundusBHV'	0.203802
Artibeus_lituratus	'ArtibeuslituratusLR4BHV'	0.22443
Mus_musculus	'U68299.1MouseCMV1'	0.209756
Rattus_colletti	'AF232689.2Ratcytomegalovirus'	0.209756
Macaca_fascicularis	'JN227533.1CynomolgusmacaqueCMV'	0.134237
Macaca_mulatta	'AY186194.1RhesusCMV'	0.134237
Papio_hamadryas	'KR351281.1PapioursinusCMV'	0.13641
Pan_troglodytes	'AF480884.1PanineHV2'	0.13497
Gorilla_gorilla	'KX839487.1GorillaberingeiberingeiCMV1'	0.134213
Homo_sapiens	'KP745648.1HumanHHV5'	0.135307
Aotus_azarai	'FJ483970.2AotineHV1'	0.166036
Plecotus_austriacus	'JX294562.1PlecotusaustriacusBHV2'	0.067605
Nyctalus_noctula	'JX294556.1NyctalusnoctulaBHV1'	0.068057
Pipistrellus_pipistrellus	'JX294558.1PipistrelluspipistrellusBHV1'	0.068058
Rousettus_egyptiacus	'JX294566.1RousettusaegyptiacusBHV2'	0.260015

Host	Virus (amino acid sequence)	Jackknifed squared residuals
Rattus_colletti	'AVI45184.1RodentHV'	0.09721
Mus_musculus	'YP_007016486.1MuridBHV8'	0.09721
Gorilla_gorilla	'KX839487.1GorillaberingeiberingeiCMV1'	0.01793
Pan_troglodytes	'NP_612722.1PanineBHV2'	0.0175
Homo_sapiens	'AAC40814.1HumanHHV5'	0.01519
Macaca_fascicularis	'YP_009337529.1CynomolgusCMV'	0.03499
Aotus_azarai	'YP_004940106.1AotineBHV1'	0.07857
Miniopterus_schreibersi	'EF151197.1MiniopterusschreibersiiBHV1'	0.04926
Tadarida_teniotis	'JX294564.1TadaridateniotisBHV2'	0.04813
Myotis_oxyotus	'MyotisoxyotusCAJ1BHV'	0.03667
Plecotus_austriacus	'JX294562.1PlecotusaustriacusBHV2'	0.03756
Pipistrellus_pipistrellus	'JX294558.1PipistrelluspipistrellusBHV1'	0.04254
Nyctalus_noctula	'JX294556.1NyctalusnoctulaBHV1'	0.04254
Rhynchonycteris_naso	'RhynchonycterisnasoLR4BHV'	0.12948
Saccopteryx_bilineata	'SaccopteryxbilineataLR4BHV'	0.12948
Sturnira_tildae	'SturniraLR4BHV'	0.02524
Artibeus_lituratus	'ArtibeuslituratusLR4BHV1'	0.0339
Desmodus_rotundus	'DesmodusrotundusBHVfullgenome'	0.08832
Glossophaga_soricina	'GlossophagasoricinaLR1BHV'	0.0512
Rousettus_egyptiacus	'AGK92605.1RousettusaegyptiacusBHV1'	0.06699
Cavia_porcellus	'YP_007417856.1CaviidBHV2'	0.25987
Diphylla_ecaudata	'DiphyllaecuadataBHV'	0.46683
Tupaia_picta	'NP_116438.1TupaiidBHV1'	0.14837

Supplementary Table 5 – Bat saliva samples selected for metagenomic sequencing.

Sample ID	10144_KF28	10144_KF29	10148_KF28	10148_KF29
Bat ID	10144	10144	10148	10148
Department	Cusco	Cusco	Cusco	Cusco
Age	Adult	Adult	Sub-adult	Sub-adult
Sex	Male	Male	Female	Female
Year of collection	2018	2018	2018	2018
Sample treatment	None	DNase	None	DNase
Raw reads	42,266,639	48,871,429	33,079,026	47,376,739
Putative viral reads	175,802	259,501	421,307	1,116,180
Reads mapped uniquely to DrBHV	5,705	12,757	56,781	154,076
Percentage of viral reads	3	5	13	14
Average coverage when aligned to the DrBHV consensus genome (reads/nucleotide)	3.70	8.28	36.87	100.05

Supplementary Table 6 – Primers for vampire bat rabies virus RT-PCR and sequencing

Name	Sequence
001F	ACG CTT AAC AAC AAA AYC
1152R	TCA GAA TTG ACT GTT CCG TC
304R	TTG ACA AAG ATC TTG CTC AT
471F	ACC GGC AAT TAC AAG ACA AAC
550DegR	GGT ACT CCA GTT AGC RCA CAT
930F	CCT CAT TCA CTT TGT TGG ATG

Supplementary Table 7 – Sequences used for BHV phylogenetic analysis.

Sequence	Link
KY290185/Homo_sapien/HHV6	https://www.ncbi.nlm.nih.gov/nuccore/KY290185
KP745648.1/Homo_sapien/HHV_5	https://www.ncbi.nlm.nih.gov/nuccore/KP745648.1
KX839487.1/Gorilla_beringei_beringei/CMV_1	https://www.ncbi.nlm.nih.gov/nuccore/KX839487.1
KX839485.1/Gorilla_beringei_beringei/CMV_2	https://www.ncbi.nlm.nih.gov/nuccore/KX839485.1
AF480884.1/Panine/BHV_2	https://www.ncbi.nlm.nih.gov/nuccore/AF480884.1
FJ483970.2/Aotine/BHV_1	https://www.ncbi.nlm.nih.gov/nuccore/FJ483970.2
KX689268.1/Macacine/BHV_3	https://www.ncbi.nlm.nih.gov/nuccore/KX689268.1
AY536265.1/Cercopithecine/HV_8	https://www.ncbi.nlm.nih.gov/nuccore/AY536265.1
JN227533.1/Cynomolgus_macaque/CMV	https://www.ncbi.nlm.nih.gov/nuccore/JN227533.1
HG531783.1/Caviid/BHV_2	https://www.ncbi.nlm.nih.gov/nuccore/HG531783.1
AF281817.1/Tupaiid/BHV_2	https://www.ncbi.nlm.nih.gov/nuccore/AF281817.1
U68299.1/Mouse/CMV_1	https://www.ncbi.nlm.nih.gov/nuccore/U68299.1
AF232689.2/Rat/CMV	https://www.ncbi.nlm.nih.gov/nuccore/AF232689.2
JX294564.1/Tadarida_teniotis/BHV_2	https://www.ncbi.nlm.nih.gov/nuccore/JX294564.1
EF151197.1/Miniopterus_schreibersii/BHV_1	https://www.ncbi.nlm.nih.gov/nuccore/EF151197.1
JX294566.1/Rousettus_aegyptiacus/BHV_2	https://www.ncbi.nlm.nih.gov/nuccore/JX294566.1
KT886845.1/Myotis_escalerai/BHV_2	https://www.ncbi.nlm.nih.gov/nuccore/KT886845.1
JX294560.1/Pipistrellus_pygmaeus/BHV_1	https://www.ncbi.nlm.nih.gov/nuccore/JX294560.1
JX294558.1/Pipistrellus_pipistrellus/BHV_1	https://www.ncbi.nlm.nih.gov/nuccore/JX294558.1
JX294559.1/Pipistrellus_pipistrellus/BHV_2	https://www.ncbi.nlm.nih.gov/nuccore/JX294559.1
JX294556.1/Nyctalus_noctula/BHV_1	https://www.ncbi.nlm.nih.gov/nuccore/JX294556.1
JX294562.1/Plecotus_austriacus/BHV_2	https://www.ncbi.nlm.nih.gov/nuccore/JX294562.1

Supplementary Figure 1 – Schematic representation of the DrBHV genome

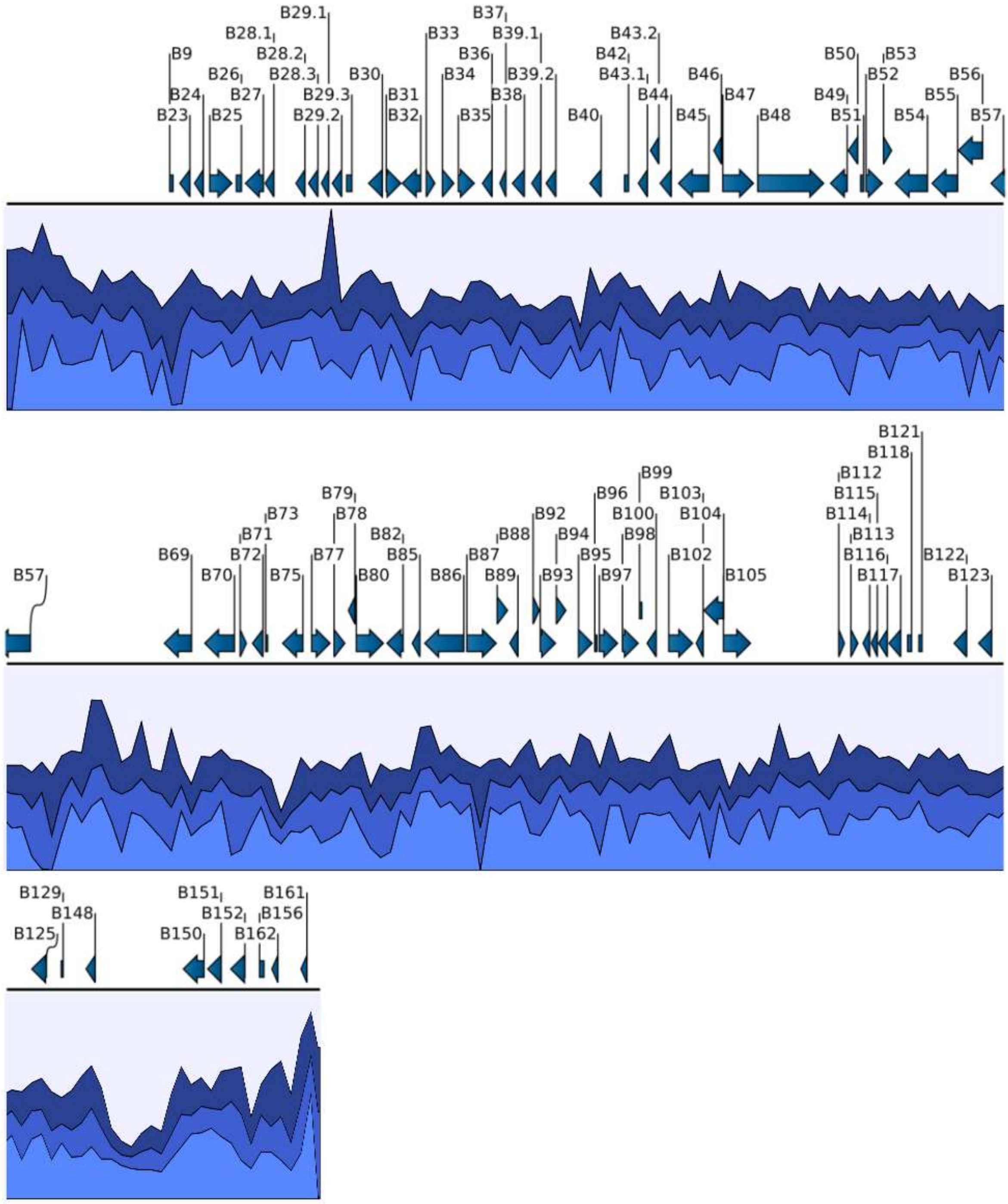

Schematic representation of the DrBHV genome with gene annotation in relation to *Miniopterus schreibersii* BHV B7D8 proteins (top part of each panel) and coverage by sequencing reads from sample 10148_KF29 (bottom part). In the graph, dark, mid- and light blue shading represent maximum, mean and minimal coverage depth values.

Supplementary Figure 2 - Haplotype network of samples 10148 and 10144 illustrating super-infection and intra-host evolution.

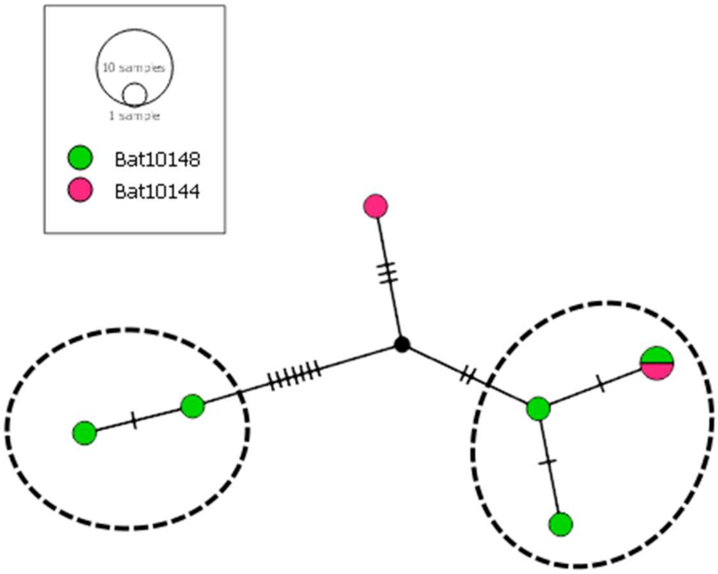

Tight span walker network of haplotypes found at position 206,389-206,438 of samples 101048 (green) and 10144 (pink). The number of mutations between each haplotype is represented by hatch marks. The dotted rings highlight putative separately introduced strains for bat 10148, from which within host evolution is taking place. Network created in PopART (http://popart.otago.ac.nz).
