# Supplementary material for: Epidemiology and biology of a herpesvirus in rabies endemic vampire bat populations
Source: Nat Commun. 2020 Nov 23;11:5951. doi: 10.1038/s41467-020-19832-4 (PMC7683562; doi:10.1038/s41467-020-19832-4)
Supplement: Supplementary file 3 — Description of Additional Supplementary Files [file 41467_2020_19832_MOESM3_ESM.pdf]

Description of Additional Supplementary Files

File Name: Supplementary Data 1
Description: Full comparison of individual vampire bats tested by BHV PCR, RV RT-PCR and RV serology.

File Name: Supplementary Data 2
Description: DrBHV whole genome sequence 5000bp sections Blast results table.
