# Supplementary material for: Epidemiology and biology of a herpesvirus in rabies endemic vampire bat populations
Source: Nat Commun. 2020 Nov 23;11:5951. doi: 10.1038/s41467-020-19832-4 (PMC7683562; doi:10.1038/s41467-020-19832-4)
Supplement: Supplementary file 6 — Reporting Summary [file 41467_2020_19832_MOESM6_ESM.pdf]

nature research

Corresponding author(s): Megan Griffiths

Last updated by author(s): Oct 13, 2020

Reporting Summary

Nature Research wishes to improve the reproducibility of the work that we publish. This form provides structure for consistency and transparency in reporting. For further information on Nature Research policies, see our Editorial Policies and the Editorial Policy Checklist.

Statistics

For all statistical analyses, confirm that the following items are present in the figure legend, table legend, main text, or Methods section.

n/a	Confirmed	
☐	☒	The exact sample size (*n*) for each experimental group/condition, given as a discrete number and unit of measurement
☐	☒	A statement on whether measurements were taken from distinct samples or whether the same sample was measured repeatedly
☐	☒	The statistical test(s) used AND whether they are one- or two-sided<br>*Only common tests should be described solely by name; describe more complex techniques in the Methods section.*
☐	☒	A description of all covariates tested
☐	☒	A description of any assumptions or corrections, such as tests of normality and adjustment for multiple comparisons
☐	☒	A full description of the statistical parameters including central tendency (e.g. means) or other basic estimates (e.g. regression coefficient) AND variation (e.g. standard deviation) or associated estimates of uncertainty (e.g. confidence intervals)
☐	☒	For null hypothesis testing, the test statistic (e.g. *F*, *t*, *r*) with confidence intervals, effect sizes, degrees of freedom and *P* value noted<br>*Give P values as exact values whenever suitable.*
☒	☐	For Bayesian analysis, information on the choice of priors and Markov chain Monte Carlo settings
☒	☐	For hierarchical and complex designs, identification of the appropriate level for tests and full reporting of outcomes
☒	☐	Estimates of effect sizes (e.g. Cohen's *d*, Pearson's *r*), indicating how they were calculated

Our web collection on statistics for biologists contains articles on many of the points above.

Software and code

Policy information about availability of computer code

Data collection	No software was used to collect the data.
Data analysis	Statistical analysis was carried out in R studio v1.1.456 (R v3.6.3), packages binom and lme4.<br>Sequences were aligned using MUSCLE (EMBL-EBI).<br>Phylogenetic inference was performed using BEASTv1.10.4 and Tracer v1.71 and visualised in FigTree v1.4.4 and R package ggtree v2.2.1.<br>Co-phylogenetic analysis was performed in R using the package paco v0.4.2 and visualised using ape v5.4-1.<br>Illumina sequence reads were cleaned and trimmed using trim_galore and diamond v0.9.25 as part of the allmond bioinformatics pipeline for viral discovery (https://github.com/rjorton/Allmond). Reads were de novo assembled using SPAdes v3.10.1 and reference assembled using bowtie2 v2.3.5.1 and bwa v0.7.17 and samtools v1.9. The sequence was annotated using GeneMarkS to identify predicted open reading frames, and CLCGenomics Workbench v7.5.1.<br>Sequences in Figure 5 were extracted using phyloscanner v1.8.0 and visualised in R using ggtree v2.2.1.<br>Supplementary Figure 2 was created using PopART (http://popart.otago.ac.nz).

For manuscripts utilizing custom algorithms or software that are central to the research but not yet described in published literature, software must be made available to editors and reviewers. We strongly encourage code deposition in a community repository (e.g. GitHub). See the Nature Research guidelines for submitting code & software for further information.

Data

Policy information about availability of data

All manuscripts must include a data availability statement. This statement should provide the following information, where applicable:

- Accession codes, unique identifiers, or web links for publicly available datasets
- A list of figures that have associated raw data
- A description of any restrictions on data availability

Results of the betaherpesvirus PCR, rabies virus RT-PCR and rabies virus neutralising antibody test, for Peruvian bats, generated and/or analysed during the current study are available in the figshare repository https://doi.org/10.6084/m9.figshare.13090214. Source data for figures 1 and 2 are provided as a source data file. UL89 partial sequences used for phylogenies have been uploaded to GenBank, with the following accession numbers: MT432305-16 and MT912480-93 (https://www.ncbi.nlm.nih.gov/nuccore/MT432305, https://www.ncbi.nlm.nih.gov/nuccore/MT432306, https://www.ncbi.nlm.nih.gov/nuccore/MT432307, https://www.ncbi.nlm.nih.gov/nuccore/MT432308, https://www.ncbi.nlm.nih.gov/nuccore/MT432309, https://www.ncbi.nlm.nih.gov/nuccore/MT4323010, https://www.ncbi.nlm.nih.gov/nuccore/MT4323011, https://www.ncbi.nlm.nih.gov/nuccore/MT4323012, https://www.ncbi.nlm.nih.gov/nuccore/MT4323013, https://www.ncbi.nlm.nih.gov/nuccore/MT4323014, https://www.ncbi.nlm.nih.gov/nuccore/MT4323015 https://www.ncbi.nlm.nih.gov/nuccore/MT4323016, https://www.ncbi.nlm.nih.gov/nuccore/MT912480, https://www.ncbi.nlm.nih.gov/nuccore/MT912481, https://www.ncbi.nlm.nih.gov/nuccore/MT912482, https://www.ncbi.nlm.nih.gov/nuccore/MT912483, https://www.ncbi.nlm.nih.gov/nuccore/MT912484, https://www.ncbi.nlm.nih.gov/nuccore/MT912485, https://www.ncbi.nlm.nih.gov/nuccore/MT912486, https://www.ncbi.nlm.nih.gov/nuccore/MT912487, https://www.ncbi.nlm.nih.gov/nuccore/MT912488, https://www.ncbi.nlm.nih.gov/nuccore/MT912489, https://www.ncbi.nlm.nih.gov/nuccore/MT912490, https://www.ncbi.nlm.nih.gov/nuccore/MT912491, https://www.ncbi.nlm.nih.gov/nuccore/MT912492, https://www.ncbi.nlm.nih.gov/nuccore/MT912493). The VBRV sequence from bat 6024 has the accession number MT891038 (https://www.ncbi.nlm.nih.gov/nuccore/MT891038,). The chimeric consensus sequence and aligned sequence reads have been submitted to the SRA with bioproject ID: PRJNA631425, and run IDs, SRR11789719-20 (https://trace.ncbi.nlm.nih.gov/Traces/sra/?run=SRR11789719, https://trace.ncbi.nlm.nih.gov/Traces/sra/?run=SRR11789720). The data used for panel G of Figure 2 can be found at https://www.senasa.gob.pe/senasa/reportes-epidemiologicos-semanales/.

Field-specific reporting

Please select the one below that is the best fit for your research. If you are not sure, read the appropriate sections before making your selection.

☐ Life sciences ☐ Behavioural & social sciences ☒ Ecological, evolutionary & environmental sciences

For a reference copy of the document with all sections, see nature.com/documents/nr-reporting-summary-flat.pdf

Ecological, evolutionary & environmental sciences study design

All studies must disclose on these points even when the disclosure is negative.

Study description	Saliva, blood and serum samples were collected from wild bat (predominantly common vampire bat) colonies in order to determine the presence of betaherpesviruses and characterise those found including prevalence, genome sequencing and phylogenetics.
Research sample	The research sample consisted of common vampire bats (Desmodus rotundus) captured at 37 sites across Peru. Individuals were male and female bats of adult, sub-adult, and juvenile age. This species is the most appropriate for the research question posed which involved a transmissible viral vector for a vampire bat rabies vaccine. The colonies we studied are of typical size, age and sex distributions for this species in Peru. The research sample is therefore designed to apply more broadly to other populations of this species. The research sample also included 19 other species of bats native to Peru which co-roost with D. rotundus in order to assess the phylogenetics and cross species transmission of virus: Artibeus fraterculus, Artibeus planirostris, Artibeus obscurus, Artibeus lituratus, Chiroderma trinitatum, Chiroderma salvini, Vampyressa bidens, Sturnira lilium, Carollia perspicillata, Rhinophylla pumilio, Choeroniscus minor Anoura geoffroyi, Glossophaga soricina, Phyllostomus discolor, Lonchorhina aurita, Diphylla ecaudata, Myotis oxyotus, Saccopteryx bilineata and Rhynchonycteris naso.
Sampling strategy	Sample sizes for field studies depended on capture success of each species of bat, which was largely beyond our control. However, a wide range of bat colonies were selected in order to sample multiple bat species. Vampire bat only colonies were also sampled as this species was most relevant for the study. Sites across Peru were used in order to observe any regional differences. These samples were drawn from a long term research monitoring programme in Peru. sample sizes were sufficient to detect DrBHV in all sampled populations. For other bat species, greater sampling was not possible and (as discussed in the manuscript) likely limited our ability to detect other viruses and accurately assess their prevalence. For rabies serology and molecular assays, sample sizes were based on previous studies showing antibody prevalence ~10% and shedding incidence of <1% and our sample sizes were sufficient to detect both. No statistical analysis was performed on those data.
Data collection	Field data were collected predominately by co-author Carlos Tello, with some assistance from other co-authors (Becker, Streicker, Bergner). Saliva, fecal and blood samples were collected from captured bats. Saliva swab samples were collected by allowing bats to chew on sterile cotton-tipped wooden swabs (Fisherbrand) for 10 s. Fecal samples were collected by using a sterile cotton swab to sample the anus. Whole blood samples were also collected on swabs after puncturing the propatagial vein with a sterile 23-gauge needle. Laboratory analysis of samples was carried out by Alice Broos, Laura Bergner and Megan Griffiths. Sequencing was carried out by the CVR viral sequencing unit led by Ana da Silva Filipe.
Timing and spatial scale	The data used in this study were collected between 7-July-2016 and 13-June-2018 as part of an on-going series of bat captures and data collections. No shipments of samples have been received since 2018. Some colonies were sampled in a single cross-sectional study, and others in a longitudinal study across several years, with the data from both sets combined here. Sites were spread across 8 administrative regions within Peru, with a maximum distance of approximately 1200km. This spatial scale was selected to investigate potential regional differences in virus prevalence and sequence.

Data exclusions	No data were excluded from this study.
Reproducibility	Results shown are based upon observational data, sequencing, evolutionary and bioinformatic analysis. No controlled experiments were carried out. Each of our bat colonies is considered a replicate of the incidence of DrBHV infection and all colonies had similarly high infection prevalence, indicating high reproducibility. Reproducibility of PCRs carried out in the lab was ensured by the use of positive and negative controls for each batch. Reproducibility in the phylogenies constructed was ensured by the use of MCMC sampling run for 1E7 generations, and co-phylogenetic analyses were tested for significance with 1E5 permutations. Whole genome sequencing of the two samples selected could not be repeated due to lack of sample.
Randomization	Sites were selected through a regionally stratified random sample among those available from historical sampling. The individual bats within colonies that were selected for testing were chosen at random.
Blinding	Blinding was not relevant since data were observational and no experimental classes were assigned. There were no pre-existing biases to mitigate by blinding.

Did the study involve field work? [x] Yes [ ] No

Field work, collection and transport

Field conditions	The study was carried out in Peru, in 37 sites across 8 departments. These location include sites in Andes, Amazon and coastal regions. The coastal region is largely a subtropical coastal desert with little appreciable rainfall. The Andes mountains observe a cool-to-cold climate with rainy summers and very dry winter. The amazon eastern lowlands region demonstrates an equatorial climate with hot weather and rain distributed all year long.
Location	Field samples were collected from 37 bat roosts across Peru as follows: Amazonas (AMA2, -5.2163067, -78.28258409, elevation=335m; AMA4, --5.81877, -77.82368; AMA5, -6.22235, -77.60754), Apurimac (API1, -13.44984759, -73.83016029, elevation=1989m; API140, -14.1415, -73.1402, elevation=2834m; API141, -13.7892, -72.8546, elevation=2468m; API15, -13.4913, -73.8103, elevation=2105m; API17, -14.0845, -72.0618, elevation=2973m; API18, -13.9229, -73.1904, elevation=2990m; API13, -13.65346304, -72.91623278, elevation=2090m; API9, -13.58511052, -73.352477, elevation=3061m), Ayacucho (AYA1, -13.041, -73.9571, elevation=2529m; AYA11, -13.4238, -73.8419, elevation=2042m; AYA12, -13.042, -73.9617, elevation=2493m; AYA14, -13.9293, -73.9123, elevation=2982m; AYA15, -13.4263, -73.8662, elevation=2283m; AYA4, -13.025, -73.9106, elevation=2736m; AYA5; AYA7, -13.23195158, -73.628092, elevation=3295m), Cajamarca (CAJ1, -6.46456, -78.67991, elevation=2882m; CAJ2, -6.40509, -78.85663, elevation=2626m; CAJ3, -6.4017, -78.77091, elevation=2597m; CAJ4, -5.16892, -78.9539, elevation=788m), Cusco (CUS6, -13.65257, -72.24618, elevation=2870m; CUS8, -13.4917, -72.508, elevation=2486m), Huanuco (HUA1, -9.19178, -75.9579, elevation=711m; HUA2, -10.2229, -76.2575, elevation=2342m; HUA4, -9.11973, -76.0065, elevation=696m), Lima (LMA10, -11.5883, -77.2784, elevation=2m; LMA12, -12.1833, -76.85, elevation=202m; LMA4, -12.6656, -76.668, elevation=7m; LMA5, -10.6415, -77.816, elevation=66m; LMA6, -11.0555, -77.4594, elevation=354m) and Loreto (LR1, -4.24127, -73.3029; LR2, -4.29033, -73.1986; LR3, -4.30665, -73.2185; LR4, -4.21052, -73.2044).
Access & import/export	Samples were collected under the Peruvian collection permits: RD-009-2015-SERFOR-DGGSPFFS, RD-264-2015-SERFOR-DGGSPFFS, RD-142-2015-SERFOR-DGGSPFFS, RD-054-2016-SERFOR-DGGSPFFS.
Disturbance	Disturbance to bats was minimised by holding bats in individual cloth bags for the minimum time required to process them (generally <2h). Disturbance to field sites was minimal, potentially including minor disturbance around roosts where nets were set up. All animals were released after sampling at the sites of capture.

Reporting for specific materials, systems and methods

We require information from authors about some types of materials, experimental systems and methods used in many studies. Here, indicate whether each material, system or method listed is relevant to your study. If you are not sure if a list item applies to your research, read the appropriate section before selecting a response.

Materials & experimental systems

n/a	Involved in the study
[x]	[ ] Antibodies
[x]	[ ] Eukaryotic cell lines
[x]	[ ] Palaeontology and archaeology
[ ]	[x] Animals and other organisms
[x]	[ ] Human research participants
[x]	[ ] Clinical data
[x]	[ ] Dual use research of concern

Methods

n/a	Involved in the study
[x]	[ ] ChIP-seq
[x]	[ ] Flow cytometry
[x]	[ ] MRI-based neuroimaging

Animals and other organisms

Policy information about studies involving animals; ARRIVE guidelines recommended for reporting animal research

Laboratory animals	The study did not involve laboratory animals.

Wild animals	Wild common vampire bats (Desmodus rotundus) and other bat species listed above were captured in Peru. Both males and females of adult, subadult and juvenile age were sampled. Bats were captured using hand nets within roosts during the day and using mist nets to capture bats exiting to forage at night. Nocturnal captures lasted from approximately 18:00 – 6:00, and nets were checked every 30 minutes. Animals were held in individual bags prior to processing, and were released in the same area where they were captured immediately after sampling.
Field-collected samples	No laboratory work was performed with field collected animals. Animals were released at the site of capture after sampling. Saliva, serum and blood samples were collected from bats and analysed by PCR.
Ethics oversight	All capture and sampling of bats was approved by the Research Ethics Committee of the University of Glasgow School of Medical, Veterinary and Life Sciences (Ref081/15) and by the University of Georgia Animal Care and Use Committee (A2014 04-016-Y3-A5).

Note that full information on the approval of the study protocol must also be provided in the manuscript.
